# Tocilizumab treatment in MOGAD: a case report and literature review

**DOI:** 10.1007/s10072-023-07189-7

**Published:** 2023-11-27

**Authors:** Giuseppe Schirò, Salvatore Iacono, Michele Andolina, Alessia Bianchi, Paolo Ragonese, Giuseppe Salemi

**Affiliations:** 1 Department of Biomedicine, Neurosciences and Advanced Diagnostics, University of Palermo, Palermo, Italy; 2 Neurology Unit, Department of Diagnostic and Therapeutic Radiology & Stroke, AOU Policlinico, Palermo, Italy; 3 Queen Square MS Centre, Department of Neuroinflammation, Institute of Neurology, Faculty of Brain Science, University College London, London, UK

**Keywords:** Tocilizumab, MOGAD, Demyelinating disorder, Leukodystrophy, COVID-19

## Abstract

Myelin oligodendrocyte glycoprotein-immunoglobulin G associated disease (MOGAD) is an autoimmune demyelinating disorder of the central nervous system (CNS) which usually occurs with recurrent optic neuritis, transverse myelitis, acute disseminating encephalomyelitis, or brainstem encephalitis. To date, the anti-CD 20 drug rituximab (RTX) is employed in MOGAD although some authors reported the efficacy of Tocilizumab (TCZ) in refractory patients. We present the case of a woman affected by refractory MOGAD who was treated with TCZ after therapy with RTX had failed to prevent relapses. We also conducted a current literature review on TCZ use in MOGAD. A 57-year-old Caucasian woman affected by MOGAD with severe motor impairment and cognitive dysfunction was treated from 2020 to February 2022 with RTX. However, she experienced progressive clinical and cognitive worsening associated with white matter lesions mimicking leukodystrophy. In February 2022, the patient started therapy with TCZ administered with improvement of cognitive performance, walking ability, and brainstem functions. During TCZ, our patient reached the condition of NEDA-3 (no relapse, no increase in disability, no MRI activity on neuroimaging follow-up performed in September 2023). Moreover, the patient experienced paucisymptomatic SARS-CoV-2 infection that did not modify TCZ schedule. To date, there are few evidence on the efficacy and safety of TCZ in MOGAD. However, all the reviewed cases showed that TCZ represents an effective therapy in drug-resistant MOGAD. Our case highlights the efficacy of TCZ in drug resistant MOGAD and strengthens previous reports of TCZ safety and efficacy in MOGAD.

## Introduction

MOGAD is a demyelinating disease of the CNS with clinical, demographic, and radiological features different from multiple sclerosis (MS) and from aquaporin-4 (AQP4) autoantibody-associated neuromyelitis optica spectrum disorder (NMOSD). MOG is a glycoprotein present in the outer layer of the myelin sheath and on the surfaces of oligodendrocytes, probably with a structural function of adhesion protein. If the AQP4-NMOSD is an example of astrocytopathy, since the AQP4-IgG damage astrocytes, the MOGADs can be considered an example of oligodendrocytopathy [1]. Patients with MOGAD generally have a good response to immunotherapy. Traditional disease-modifying therapies (DMTs) used for MS appear ineffective or even detrimental to treat MOGAD. Immunotherapy for MOGAD includes rituximab (RTX), an anti-CD20 monoclonal antibody, azathioprine, mycophenolate mofetil, and intravenous immunoglobulins [2]. A recent meta-analysis underlined the safety and efficacy profile of RTX for relapse prevention in patients with MOGAD [3]. However, some patients with MOGAD experience a recurrence of clinical relapses and new radiological lesions despite being on RTX therapy. Recently, the efficacy and safety of tocilizumab (TCZ), a humanized antibody directed against the IL-6 receptor, has been documented in several case reports of MOGAD unresponsive to RTX as shown below in Table 1.

**Table 1:** Off-label use of TCZ: case reports and case series in MOGAD

Type of study and Number of the patients	Sex, median age at TCZ starting	Follow-up	Previous therapies	Clinical outcome	TCZ: route and dose	MRI follow-up	Adverse events	References
Case report	M, 20 years	2 years	RTX	Absence of clinical relapses	Intravenous TCZ 8 mg/kg every 4 weeks	Reduction of cervical and thoracic lesions	No adverse effects observed	[7]
Case report	M, 59 years	4.5 years	Natalizumab, RTX and ciclophosphamide	Absence of clinical relapses	IntravenousTCZ 8 mg/kg every 6 weeks	Stable MRI load	No adverse effects reported	[8]
Case series, 3 patients	2 F, 1 M, 35,6 years	18 months	RTX	Absence of clinical relapses	Intravenous TCZ 8 mg/kg per month and/or subcutaneous 162 mg per week	Stable MRI load	Tooth infections in one patient, hypertriglyceridemia in another patient, none in the third patient	[9]
Case report	F, 31 years	13 months	RTX	no data available	Intravenous TCZ 8 mg/kg	No data available	No adverse effects reported	[10]
Case series, 14 patients	5 F, 9 M, 38.4 years	Median follow-up was 16.3 months	RTX, azathioprine, mycophenolate mofetil, cyclophosphamide, belimumab, methotrexate	Absence of clinical relapses and reduction of median EDSS	Intravenous TCZ 8 mg/kg per month	MRI relapses during TCZ therapy in one patient, relapses in 4 patients	No adverse effects reported	[6]
Case series, 2 patients	2 F, 25.5 years	Approximately 24 months	Corticosteroids and RTX	Absence of clinical relapses	Intravenous TCZ 8 mg/kg per month or subcutaneous TCZ 162 mg per week	Stable MRI load	No adverse effects observed	[11]
Case report	F, 43 years	3 months	Corticosteroids and plasma exchange	Absence of clinical relapses	Intravenous TCZ 8 mg/kg	Improvement of symptoms and MRI findings	No adverse effects reported	[12]
Case report	F, 63 years	18 months	Corticosteroids, methotrexate and RTX	Absence of clinical relapses	Intravenous TCZ 400 mg/week	No clinical relapses	No adverse effects reported	[13]

**Fig. 1:**
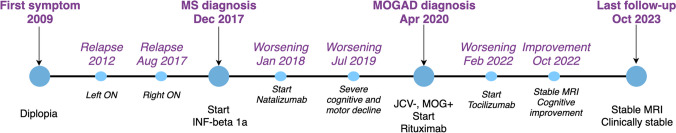
Timeline of the clinical history of the described patient

Disease pathology is linked to macrophages retrieval, microglial activation, and inflammation mediators like IL-6. IL-6, a pleiotropic cytokine upregulated in MOGAD induction and relapses, causes Th17 lymphocyte differentiation which are responsible for direct demyelination and creates positive feedback for IL-6 release [4]. MOGAD pathogenesis could be only partially supported by complement via classic activation (antibody-MOG binding). NMOSD AQP4 + antigen–antibody binding is monovalent, providing a massive activation of C1q, while in MOGAD a bivalent antibody binding is needed [5]. Hence, complement inactivation therapies did not show encouraging results in patients with MOGAD, in an opposite way to NMOSD AQP4 + individuals. For this reason, TCZ is even more frequently used in MOGAD. TCZ has been also used in complicated SARS-Cov-2 infection with hyperinflammatory syndrome to suppress cytokine release syndrome and to reduce risk for invasive mechanical ventilation or mortality. In this article, we reported the case of a relapsing MOGAD characterized by the accumulation of confluent and bilateral hemispheric lesions related to recurrent episodes of encephalitis resulting in a final radiological picture resembling a leukodystrophy pattern, refractory to anti-CD-20 therapy, who later experienced SARS-CoV-2 infection during TCZ treatment. We also reviewed and updated the existing evidence up to October 2023 on the use of TCZ in patients with MOGAD (Fig. 1).

## Clinical scenario

**Fig. 2:**
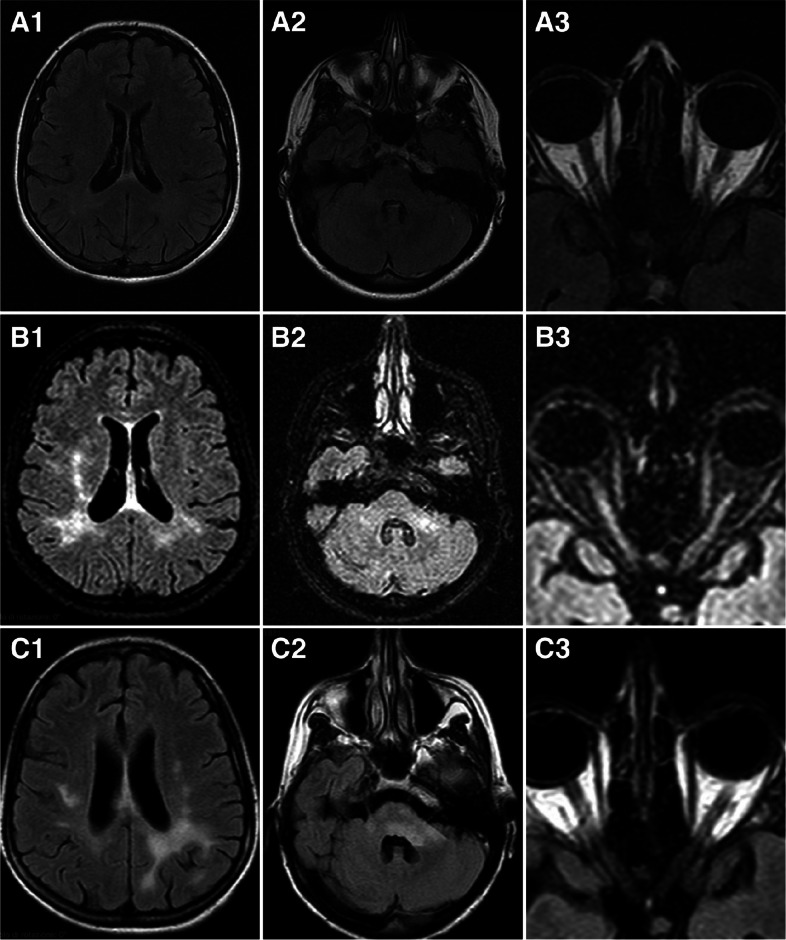
**A** MRI performed in 2012 showing the right temporo-occipital white matter hyperintensity in the axial T2-FLAIR WI (A1), the absence of lesion at infratentorial level documented in the axial T2-FLAIR-WI (A2) and the focal hyperintensity of the left optic nerve in the axial T2-FLAIR WI (A3). **B** MRI performed in 2017 showing the increasing of whiter matter hyperintensity involving the parieto-occipital lobes especially the right white matter in the axial T2-FLAIR WI (B1), the focal hyperintensity of the left middle cerebellar peduncle in the axial T2-FLAIR WI (B2) and the bilateral hyperintensity of optic nerves the axial T2-FLAIR WI (B3). **C** MRI performed in July 2018 showing the reduction of hyperintensity in right temporo-occipital white matter and the contemporary increase of hyperintensity on the left temporo-occipital white matter in the axial T2 FLAIR WI (C1), the increase of hyperintensity in the left middle cerebellar peduncle in the axial T2-FLAIR WI (C2) and the partial persistence of the hyperintensity in the bilateral optic nerves in the axial T2-FLAIR WI (C3)

**Fig. 3:**
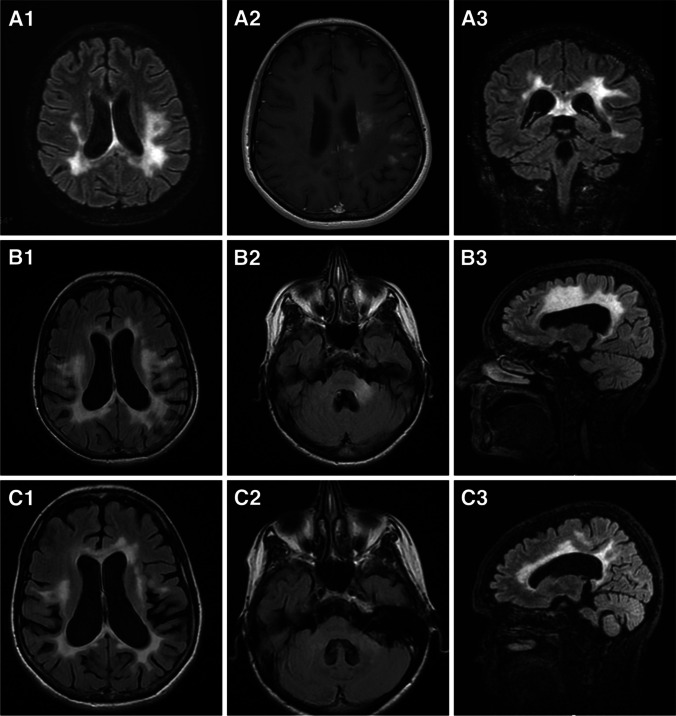
**A** MRI performed in 2019 showing the bilateral hyperintensity in the frontal and parietal white matter in the axial T2-FLAIR WI (A1), a focal and irregular post-contrast enhancement in the left periventricular white matter and parieto-occipital region in the axial T1-WI (A2) and the involvement of isthmus of corpus callous as well as the left frontal white matter in the coronal T2-FLAIR WI (A3). **B** MRI performed in January 2022 showing the extension of the bilateral hyperintensity involving the periventricular regions as well the parieto-occipital lobes in the axial T2-FLAIR WI (B1), the focal hyperintensity of the left middle cerebellar peduncle in the axial T2-FLAIR WI (B2) and the massive involvement of frontal deep and periventricular white matter in sagittal T2 FLAIR WI (B3). **C** MRI performed in October 2022 showing the reduction of hyperintensity in the axial T2 FLAIR WI (C1), the partial reduction of the hyperintensity in the left middle cerebellar peduncle in the axial T2-FLAIR WI (C2) and the partial regression of the hyperintensity in the right middle superior frontal gyrus in the sagittal T2-FLAIR WI (C3). In September 2023, the MRI was repeated and resulted stable in comparison to that performed in October 2022

In 2017, a 56-year-old Caucasian woman presented with a history of recurrent episodes of visual loss, diplopia, and gait abnormality initially followed by full recovery. Clinical examination showed brisk tendon reflexes in both lower and upper limbs as well as nystagmus and mild ataxia with a calculated EDSS of 2.0. The patient underwent lumbar puncture and brain MRI with gadolinium administration that resulted, respectively, in the absence of oligoclonal bands and bilateral demyelinating lesions of frontal, temporal, parietal white matter, and other lesions in the cerebellum, midbrain, at the level of the cerebellar peduncles, at the bulbar-medullary junction, and in the medulla at the level of D5 (Fig. 2). Conus medullaris involvement nor perineural optic sheath enhancement was reported at that time. Given the relapsing–remitting course and the findings at brain MRI, a MS diagnosis was initially made. The patient started therapy with interferon-beta-1a until July 2018, when she presented a new relapse characterized by diplopia and postural imbalance. Then, the patient started natalizumab treatment until July 2019 when she presented with severe cognitive impairment (i.e., executive functions, verbal memory, and reduced verbal fluency, spatial and temporal disorientation) and limb ataxia together with severe motor impairment. A new lumbar puncture was performed for the suspicion of progressive multi-focal leukoencephalopathy (PML), giving negative results in July 2019 and the same was repeated in October 2019 with a consistent result. A new brain MRI revealed confluent demyelinating lesions involving the parietal, temporal, and occipital white matter, some of them showing a patchy gadolinium enhancement. A MOGAD encephalitis was at that time confirmed after the presence of anti-MOG antibodies had been shown by a fixed cell-based assay in December 2019 and in March 2020. The patient underwent plasma exchange therapy after steroid therapy had failed, without significant clinical improvement. A new brain MRI showed a widespread leukoencephalopathy pattern with a post-contrast T1-weighted imaging enhancement. For this reason, in April 2020, the patient started therapy with RTX 1000 mg every 2 weeks followed by new courses every 6 months. Meanwhile, the patient became unable to walk and showed severe dysphagia and further progressive worsening of the cognitive dysfunction being unable to perform any cognitive assessment. In January 2022, there was a sudden symptom worsening with spatial agnosia, prosopagnosia, and pseudobulbar syndrome. She was admitted again to our clinic and submitted to a new course of plasma exchange, with no significant clinical improvement. A new brain MRI (Fig. 3) showed a further increase in the lesion burden both at the brain and brainstem level. Thus, in February 2022, therapy with TCZ was started at the dosing of 8 mg/kg every 4 weeks (weight 40 kg; total dose: 320 mg per infusion). IL-6 levels determined before starting therapy were 15 pg/mL (normal value < 7 pg/mL). The infusions were well tolerated; no adverse events or life-threatening conditions were reported. On April 25, 2022, despite being fully vaccinated (three doses), the patient tested positive for SARS-CoV-2 and reported fever, cough, and dyspnea. She underwent therapy with nirmatrelvir/ritonavir obtaining a dramatic regression of all symptoms in about 24 h. COVID-19 negativity was documented by a negative test for SARS-CoV-2 on May 7, 2022, and the new TCZ course was administered on May 30th. Before this administration, new IL-6 dosing showed a reduction to 4 pg/mL. During observation until January 2023, infusions of TCZ have been administered without observing new clinical events or adverse effects. In the last clinical examination, we documented on the contrary a moderate recovery of cognitive function (MMSE = 15) and walking capacity, being the patient able to walk with bilateral assistance. Family members and the caregiver of the patient confirmed the significant improvement of general conditions; the patient is actually able to assume lunches and to drink autonomously. A new MRI of the brain performed in October 2022 showed a reduction of the lesion load at the level of the middle cerebellar peduncle and the right middle-superior frontal gyrus in the absence of gadolinium-positive lesions or new T2 lesions (Fig. 3). In February 2023, a subcutaneous catch port was inserted. However, in April 2023, the patient was hospitalized for a *Pseudomonas aeruginosa* infection of the “port-a-cath,” treated with piperacilline-tazobactam therapy and removing of the port-a-cath. On May 5th, the patient was tested positive for Sars-Cov-2 and treated with remdesivir. The patient also received her scheduled administration of TCZ on May 11, 2023, during the hospitalization. In July 2023, the route of administration of TCZ was changed to a subcutaneous route at a dosage of 162 mg every two other weeks. She received six subcutaneous administrations at the date of the last follow-up up to October. At the end of September 2023, a new MRI was repeated showing no increase in lesion load (Fig. 3). At the date of the last follow-up in October 2023, no new relapse or signs of disease progression have been documented.

## Discussion

The patient we described showed a severe pattern of leukodystrophy-like/leukoencephalopathy MOGAD unresponsive to RTX treatment who on the contrary appears to improve after TCZ therapy. In this case, TCZ was associated with a favorable safety and efficacy profile, even in the presence of COVID-19.

Previous case reports support the efficacy and safety of TCZ in patients with MOGAD unresponsive to RTX or other therapies. Twenty-three patients have been previously described receiving RTX therapy alone or other treatments. The most numerous groups of patients were that of Ringelstein and colleagues who evaluated the efficacy and long-term safety of TCZ treatment in 14 patients with MOGAD, 36 with seropositive AQP4-IgG, and 7 with seronegative NMOSD. The observation period was 23.8 months. Their analysis showed that 79% of patients with MOGAD did not have relapses during TCZ treatment. The percentage of patients with absence of relapses was higher in MOGAD patients than in patients with seropositive and seronegative NMSOD AQP4-IgG [6]. Table 1 reports all the published cases supporting the efficacy and safety of TCZ in patients with MOGAD.

Of the reported twenty-five cases, including the one described by us, men represent 48% (*n* = 12) of patients and women 52% (*n* = 13). The median age at the time of TCZ initiation was 44.3 years, and the median observation period was 20.8 months. Adverse events associated with TZC therapy were observed only in two of them. A man developed a dental infection and a woman the occurrence of TCZ-related hypertriglyceridemia needing a statin prescription. We did not observe any therapy-related adverse events in our patient. Just 4 (16%), out of the 25 patients examined, presented new relapses during therapy with TCZ, and MRI disease activity was detected just in one patient (4%) [6]. No clinical relapses or appearance of further radiological lesions were observed in our patient during treatment with TCZ. Furthermore, the follow-up MRI performed in October 2022 as abovementioned showed instead an improvement in the lesion load.

The patient reported by us had previously also used interferon-beta and natalizumab due to the former diagnosis of MS. During the treatment with these therapies as well as during RTX therapy, she continued to experience relapses and a marked worsening of clinical conditions. This is analogous with other case reports that have noted a worsening of the disease in patients with AQP4 + NMOSD receiving DMTs classically used to treat MS such as beta-interferons, fingolimod, and in particular natalizumab, with accumulation of disability and disease progression [14–16]. Kleiter and colleagues suggested testing for anti-AQP4 and anti-MOG antibodies in patients diagnosed with MS before initiating natalizumab therapy, although a percentage of patients with NMOSD could be seronegative. They also proposed that in patients not responding to natalizumab, in addition to PML or the presence of neutralizing antibodies or even to the possibility of a relapse, a missed diagnosis of NMOSD should be considered [16]. It is unknown the mechanism or the mechanisms leading to worsening of MOGAD during natalizumab. An hypothesis could be the increase in the number of antibodies-producing plasma cells. In fact, natalizumab induces an increase in the numbers of peripheral B cells and CD138 + plasma cells, which in turn might increase the title of anti-MOG-antibodies that correlate with the severity of the disease. This mechanism had already been hypothesized by other authors [16] for patients with AQP4 + NMO who worsened during therapy with natalizumab. Radiological phenotypes of MOGAD are various and heterogeneous. In our patient, accumulation of lesions over time resulted in a final radiological picture mimicking that of a leukodystrophy. Leukodystrophy-like lesions are one possible presentation of MOGAD. These are very rare forms, typical of the progressive forms of MOGAD and described in pediatric patients, are associated with a poor prognosis, severe cognitive impairment, and psychiatric manifestations, and are also characterized by a poor response to common immunotherapies. In 2018, Hacohen and colleagues reported a leukodystrophy-like MRI pattern in seven pediatric MOGAD patients younger than 7 years of age [17]. Similar case reports were reported exclusively in children until in 2021, when Wang and collaborators observed a leukodystrophy-like pattern also in two adult patients with MOGAD [18]. We identified some differences between Hacohen’s pediatric cases, Wang’s two adult patients, and the adult patient we reported. Pediatric patients in Hacohen’s study had acute/subacute onset with rapid symptom progression and had a distinct clinical benefit from steroids. Contrast enhancement was observed in leukodystrophy-like lesions on MRI; differently, the two adult patients in Wang’s study experienced a chronically progressive course with no acute attacks, no benefit from steroids, and no contrast enhancement in leukodystrophy-like lesions. Finally, our patient had an acute onset of cognitive, motor, and ataxic symptoms after 11 infusions of natalizumab, developing a rapidly progressive form of dementia with subsequent slow progression of cognitive symptoms, alternating subacute phases of relapses. She did not benefit from steroids like Wang’s adult patients and occasionally had contrast enhancement in leukodystrophy-like lesions similarly to the pediatric patients.

In leukodystrophy-MOGAD, a differential diagnosis between MOGAD and leukodystrophy could be challenging. The confirmed presence of anti-MOG antibodies, especially if determinate by a cell-based assay, a history of recurrent optic neuritis, progressive cognitive and psychiatric manifestations, observation of spinal cord lesions, absence of positive familiar history, and genetic screening with negative results for leukodystrophies when necessary, is elements in favor of MOGAD. Moreover, our MRIs documented lesions of cortical regions and subcortical U-fibers, areas usually preserved in patients with leukodystrophies [19, 20].

TCZ was found to be safely administered also in the occurrence of a SARS-Cov-2 infection according to previous reports indicating how both in MOGAD and during COVID-19 there is an increase in pro-inflammatory cytokines including IL-6. Elevated levels of IL-6, expression of the cytokine release syndrome, are associated with a complicated and severe course of COVID-19 [21]. In fact, IL-6 promotes pulmonary fibrosis and respiratory dysfunction and can also inducer intrarenal inflammation, myocardial fibrosis, and kidney and gastrointestinal damage. The 8 mg/kg TCZ dosage used to treat COVID-19 pneumonia is the same administered to treat MOGAD [22, 23]. Therefore, it appears evident that treatment with TZC since it acts on a common target to the pathogenesis of COVID-19 and MOGAD, as suggested by an analogous previous case report [10], should not be stopped in case of SARS-Cov-2 infection and can be considered safe.

## Conclusion

A recurrent and refractory to therapy MOGAD can result in a radiological leukodystrophy-like/leukoencephalopathy pattern with poor outcome and low response to the common immunotherapies. TCZ can be considered an effective therapy for the treatment of these forms of MOGAD. Moreover, we reported for the first time in a patient with MOGAD a worsening of the disease during the treatment with natalizumab that showed ineffectiveness to prevent relapses. Finally, TCZ can also be considered a safe therapy, with no need for a modified schedule during Sars-Cov-2 infection.
